# The burden of palmoplantar pustulosis: A Canadian population-based study of inpatient care, emergency departments, and outpatient clinics

**DOI:** 10.1016/j.jdin.2023.04.002

**Published:** 2023-04-15

**Authors:** Jean-Eric Tarride, Vimal H. Prajapati, Charles Lynde, Gord Blackhouse

**Affiliations:** a Department of Health Research Methods, Evidence and Impact, Faculty of Health Sciences, McMaster University, Hamilton, Canada; b Programs for Assessment of Technology in Health (PATH), The Research Institute of St. Joe’s Hamilton, St. Joseph’s Healthcare, Hamilton, Canada; c Center for Health Economics and Policy Analysis (CHEPA), McMaster University, Hamilton, Canada; d Division of Dermatology, Department of Medicine, University of Calgary, Calgary, Canada; Section of Community Pediatrics, Department of Pediatrics, University of Calgary, Calgary, Canada; Section of Pediatric Rheumatology, Department of Pediatrics, University of Calgary, Calgary, Canada; Skin Health & Wellness Centre, Calgary, Canada; Dermatology Research Institute, Calgary, Canada; Probity Medical Research, Calgary, Canada; e Department of Medicine, University of Toronto, Toronto, Canada

**Keywords:** all-cause inpatient mortality, Canada, healthcare expenditures, palmoplantar pustulosis

## Abstract

**Background:**

Not much is known about the burden of palmoplantar pustulosis (PPP).

**Objectives:**

To document the burden of PPP in Canada, and to compare with psoriasis vulgaris (PV).

**Methods:**

Adult Canadians (excluding the province of Quebec) hospitalized or visiting an emergency department (ED) or hospital-/community-based clinic between April 1, 2007, and March 31, 2020, with a diagnostic code indicating PPP (ICD-10-CA: L40.3) or PV (ICD10-CA: L40.9 or L40.0) were identified using Canadian administrative data. 10-year prevalent- and 3-year incident-based approaches were conducted. Costs were determined when the most responsible diagnosis (MRD) for the admission was PPP or PV (MRD costs) and for all reasons (all-cause costs).

**Results:**

In the prevalence analysis, the 10-year mean (standard deviation [SD]) and MRD costs were $544 ($1874) for PPP and $222 ($1828) for PV (*P* < .01). In the incidence analysis, PPP patients had higher 3-year mean (SD) MRD costs ($1078 [$2705]) than PV ($503 [$2267]) (*P* < .01). All-cause costs were lower for the PPP cohort in the prevalent and incident analyses. There were no differences in all-cause inpatient mortality between PPP and PV.

**Limitations:**

Physician and prescription data were not available.

**Conclusion:**

PPP patients incurred significantly higher MRD costs than PV patients.

**Capsule Summary**

• Palmoplantar pustulosis (PPP) patients hospitalized or visiting an emergency department (ED) or a hospital-/community-based clinic due to a most responsible diagnosis (MRD) of PPP incur higher costs than patients with a MRD of psoriasis vulgaris.
• The impact of PPP beyond healthcare expenditures should be explored (quality of life, productivity).

## Introduction

Palmoplantar pustulosis (PPP), a rare subtype of pustular psoriasis, has been defined as having “primary, persistent (>3 months), sterile, macroscopically visible pustules on palms and/or soles”.^1^ Since PPP is a rare disease, the epidemiology and burden associated with it is not well understood. A recent structured review^2^ identified 4 studies reporting on the prevalence of PPP which ranged from 0.05% (Sweden)^3^ to 0.12% (Japan).^4^ To our knowledge, only 1 study,^5^ which was not included in the review by Kharawala et al^2^, attempted to capture the economic costs associated with PPP, which were found to be similar to a matched cohort of patients with psoriasis vulgaris (PV).

A recent population-based study of emergency department (ED) and inpatient care from the province of Ontario in Canada (13.6 million habitants) identified 115 ED visits and 17 hospitalizations with PPP over a 10-year period.^6^ However, this study did not provide any details on patient characteristics or on the ED visit and hospital admission (eg, length of stay and costs). To fill an important gap in the literature, the study objectives were to document the burden of PPP and to compare with PV patients.

## Methods

### Study overview and data sources

Canadian adults hospitalized and seen in an ED or hospital-/community-based outpatient clinic for either PPP (PPP cohort) or PV (PV cohort) were identified using de-identified patient-level data from 2 databases from the Canadian Institute for Health Information (CIHI)^7^: the Discharge Abstract Database (DAD) and the National Ambulatory Care Reporting System (NACRS). DAD^8^ captures administrative, demographic, and clinical information on all hospital discharges in Canada. Although NACRS coverage varies across the study years, NACRS^9^ captured approximately 65% (2016-17) to 85% (2019-20)^10^ of all hospital-/community-based ED and ambulatory care visits in Canada. All-cause inpatient or ED mortality (“inpatient mortality”) in the facility is available in DAD or NACRS while deaths available in the community are not. Data from the province of Quebec (approximately 23% of the Canadian population) were not included in this study.

Up to 20 possible International Statistical Classification of Diseases and Related Health Problems, 10th Revision, Canada (ICD-10-CA) diagnostic codes are recorded for each encounter captured in DAD and NACRS. Each diagnostic code is categorized as either a ‘most responsible diagnosis (MRD)’ (ie, the diagnosis determined to have been responsible for the greatest portion of the patient’s length of stay) or as another diagnosis/comorbidity. In addition, costs associated with each encounter in DAD and NACRS are available based on CIHI costing methodology.^11^ CIHI Meaningless but Unique Patient Identifier, which is common to DAD and NACRS, was used to link the 2 databases.

### Study population

Canadian adults (≥18 years of age) hospitalized or visiting an ED or hospital-/community-based outpatient clinic between April 1, 2017, to March 31, 2020, with a diagnostic code indicating PPP or PV were included in the study. The PPP and PV cohorts were defined by ICD-10-CA codes of L40.3 and L40.0 or L40.9, respectively. Individuals with diagnostic codes of acrodermatitis continua (ICD-10-CA code: L40.2) or dermatitis due to substances taken internally (ICD-10-CA code: L27 category, which includes generalized or local skin eruption due to drugs and medicaments taken internally) were excluded. A prevalent and an incident approach were used.

### Prevalence analysis

The number of unique patients with at least 1 DAD or NACRS record with a diagnostic code indicating PPP for the PPP cohort and PV for the PV cohort were used to estimate the number of prevalent cases over the 10-year period (April 1, 2010, to March 31, 2020). The study population was described in terms of age, sex, medical history, and several comorbidities. DAD and NACRS data were used to estimate 10-year healthcare resource utilization, costs, and all-cause inpatient mortality associated with our prevalent cohorts.

### Incidence analysis

Unique patients with a PPP or PV diagnostic code over a 5-year time period (April 1, 2012, to March 31, 2017) without any PPP (PPP incident cohort) or PV (PV incident cohort) diagnostic code in the 5 previous years (April 1, 2007, to March 31, 2012) defined our incident cases. Incident cases of PPP and PV were identified as the first DAD or NACRS record with an MRD of PPP and PV, respectively. Restricting our incident cases to those with an MRD of PPP (PPP cohort) or PV (PV cohort) was to ensure that the index event (eg, hospitalization or visit to ED or outpatient clinic) was directly attributable to PPP or PV and not to an unrelated condition. All of our 5-year incident cases had a minimum of 3-years of follow-up (up to March 31, 2020). CIHI data were used to document patient characteristics at time of index, 3-year healthcare resource utilization, costs, and all-cause inpatient mortality following the index event.

### Statistical analysis

Prevalence and incidence rates were calculated by dividing the number of prevalent/incident cases identified by the Canadian population estimates (except Quebec) during the same time period. The baseline characteristics of the PPP and PV populations were compared using student T-tests and chi-square tests. Several analyses were conducted to address some of the limitations associated with the coverage of ED and outpatient clinics captured in NACRS, which may result in an underestimate of the prevalence or incidence numbers. The first sensitivity analysis used data from Ontario only (38% of the Canadian population) as 100% of all ED visits in Ontario are reported in NACRS. The second sensitivity analysis used data from Alberta data only (12% of the Canadian population) as 100% of all ED visits and 100% of all hospital-/community-based outpatient clinics are captured in NACRS in Alberta.

The economic burden of PPP and PV was estimated using 2 approaches. DAD and NACRS records for which the MRD was PPP or PV (ie, MRD costs) were first used to identify healthcare resource utilization and costs directly attributable to PPP or PV. Our second costing approach included all health care resources consumed by the study populations independently of the presence of a PPP/PV diagnostic code (ie, all-cause costs).

Multivariable generalized linear models (GLM) (log link with negative binomial distribution for count data and gamma distribution for costs) adjusting for baseline characteristics (age, sex, and comorbidities) were used to compare the number of admissions, length of stay, and costs between the PPP and PV cohorts. Multivariable logistic regressions adjusting for baseline characteristics were used to compare all-cause inpatient mortality between PPP and PV. Cells for which between 1 and 4 individuals contributed to the data were not disclosed as this is a requirement when using CIHI data. All costs were expressed in Canadian dollars unless otherwise stated.

## Results

### Prevalence analyses

Our 10-year analysis (April 1, 2010, to March 31, 2020) identified 338 and 24,828 unique individuals with a PPP and PV record, respectively, yielding prevalence rates of 1.54 per million individuals for PPP and 113.13 per million individuals for PV. Our sensitivity analyses indicated that prevalence rates were higher in Ontario where ED visit reporting is mandatory (PPP: 1.71 per million; PV: 114.69 per million) and in Alberta where both ED visit and hospital-/community-based clinic visit reporting is mandatory (PPP: 3.45 per million; PV: 272.85 per million).

PPP patients were younger than PV patients (mean [standard deviation] of 50.9 [17.7] years and 53.3 [18.3] years, respectively; *P* = .01). There was a higher proportion of female patients in the PPP cohort (59.2%) compared to the PV cohort (44.1%) (*P* < .01). More than half of our population was from Ontario. Compared to PV patients, lower levels of comorbidities were seen among PPP patients in terms of hypertension (24.0% for PPP vs 32.9% for PV; *P* < .01), hyperlipidemia (4.4% for PPP vs 7.8% for PV; *P* = 0.02), obesity (4.1% for PPP vs 7.9% for PV; *P* = .02), thyroid disorders (3.0% for PPP vs 6.3% for PV; *P* = .01) and sleep apnea (1.8% for PPP vs 5.2% for PV; *P* < .01). Table I presents the details.

**Table I:** Prevalence analysis: baseline characteristics

	PPP	PV	*P*-value
	*N* = 338	*N* = 24,828	
Female sex	200 (59.2%)	10,954 (44.1%)	<.01
Mean age (SD)	50.9 (17.7)	53.3 (18.3)	.01
Age categories (years)			
18-25	16 (9.5%)	888 (6.8%)	.01
26-35	41 (12.1%)	3155 (12.7%)	
36-45	44 (13.0%)	3429 (13.8%)	
46-55	68 (20.1%)	4466 (18.0%)	
56-65	86 (25.4%)	4977 (20.0%)	
66-75	34 (10.1%)	3852 (15.5%)	
≥75	31 (9.2%)	3191 (12.9%)	
Province/Territory			
Alberta	109 (32.2%)	8633 (34.8%)	<.01
British Columbia	13 (3.8%)	910 (3.7%)	
Saskatchewan	7 (2.1%)	608 (2.4%)	
Manitoba	6 (1.8%)	580 (2.3%)	
Ontario	188 (55.6%)	12,609 (50.8%)	
Nova Scotia, New Brunswick, Prince Edward Island, Newfoundland, and all 3 Territories	15 (4.4%)	1488 (6.2%)	
Comorbidities			
Hypertension	81 (24.0%)	8166 (32.9%)	<.01
Diabetes	57 (16.9%)	5026 (20.2%)	.12
Cigarette smoking	24 (7.1%)	2115 (8.5%)	.35
Hyperlipidemia	15 (4.4%)	1946 (7.8%)	.02
Myocardial infarction	15 (4.4%)	1464 (5.9%)	.26
Stroke	13 (3.8%)	1060 (4.3%)	.70
Obesity	14 (4.1%)	1967 (7.9%)	.01
Sleep apnea	6 (1.8%)	1302 (5.2%)	<.01
Depression	33 (9.8%)	2810 (11.3%)	.37
Anxiety	8 (2.4%)	819 (3.3%)	.34
Thyroid disorders	10 (3.0%)	1576 (6.3%)	.01
Atopic dermatitis	ND	325 (1.3%)	.78
Gout	11 (3.3%)	1256 (5.1%)	.13
Psoriatic arthritis	8 (2.4%)	534 (2.2%)	.79

*PPP*, Palmoplantar pustulosis; *PV*, psoriasis vulgaris; *SD*, standard deviation.

As shown in Table II, PPP patients incurred higher 10-year mean (standard deviation [SD]) MRD costs than PV patients ($544 [$1874] vs $222 [$1828]; *P* < .01). However, the 10-year mean (SD) all-cause costs were lower for PPP compared to PV ($33,348 [$71,610] vs $42,574 [$83,960], respectively; *P* = .01). Over the 10-year time period, lower all-cause inpatient mortality was observed among PPP patients compared to PV patients, although this was not a statistically significant finding (3.9% vs 7.3%; *P* = 0.12). Supplementary Table I (10-year costs available via Mendeley at https://data.mendeley.com/datasets/9fnfyys96m/1) and Supplementary Table II (10-year mortality available via Mendeley at https://data.mendeley.com/datasets/9fnfyys96m/1) presents the regression results.

**Table II:** Prevalence analysis: 10-year mean (standard deviation) healthcare resource utilization and costs—most responsible diagnosis (MRD)∗ versus all-cause admissions

	Most responsible diagnosis (MRD) admissions			All-cause admissions		
	PPP (*N* = 338)	PV (*N* = 24,828)	*P*-value∗	PPP (*N* = 338)	PV (*N* = 24,828)	*P*-value†
Number of hospitalizations per case	0.08 (0.27)	0.02 (0.16)	<.01	2.25 (3.61)	2.89 (4.59)	.12
Number of hospitalizations amongst those with at least 1 hospitalization	1.00 (0.00)	1.14 (0.69)	.64	3.89 (4.02)	4.44 (5.05)	.53
Number of days in hospital for those with inpatient stay	7.30 (5.29)	6.97 (13.66)	.23	9.64 (23.22)	9.50 (21.7)	.96
Cost of hospitalization for those with inpatient stay	$5602 ($4049)	$5811 ($10,850)	.48	$10,127 ($15,841)	$10,696 ($16,894)	.60
Number of ED visits per case	0.54 (0.51)	0.44 (0.77)	.13	11.88 (16.20)	14.01 (27.79)	.08
Number of ED visits amongst those with at least 1 ED visit	1.01 (0.11)	1.18 (0.87)	.05	12.58 (16.41)	15.10 (28.57)	.03
Cost of ED visit for those with ED visit	$151 ($55)	$158 ($68)	.17	$292 ($180)	$308 ($182)	.84
Number of clinic visits per case	0.04 (0.25)	0.27(4.15)	<.01	5.17 (17.32)	9.11 (51.22)	.06
Number of clinic visits amongst those with at least 1 clinic visit	1.33 (0.71)	4.17 (15.93)	.05	16.04 (27.58)	23.65 (80.41)	.29
Cost of clinic visit for those with clinic visit	$158 ($13)	$191 ($101)	.09	$547 ($869)	$524 ($937)	.25
Number of other NACRS visits	0.03 (0.25)	0.01 (0.12)	.57	4.75 (11.34)	5.50 (15.59)	.87
Number of other NACRS visits amongst those with at least 1 other NACRS visit	1.50 (1.22)	1.22 (0.69)	.58	7.86 (13.74)	9.09 (19.21)	.92
Cost of other NACRS visit for those with at least 1 other NACRS visit	$493 ($726)	$313 ($439)	.87	$750 ($533)	$755 ($732)	.45
Total cost (1 + 2 + 3 + 4)	$544 ($1874)	$222 ($1828)	<.01	$33,348 ($71,610)	$42,574 ($83,960)	.01

*ED*, Emergency department; *NACRS*, National Ambulatory Care Reporting System; *PPP*, palmoplantar pustulosis; *PV*, psoriasis vulgaris; *SD*, standard deviation.

∗ The most responsible diagnosis (MRD) is the diagnosis (eg, PPP) determined to have been responsible for the greatest portion of the patient’s length of stay.

† *P*-values based on multivariable generalized linear models adjusting for baseline characteristics (age, sex, and comorbidities).

### Incidence analyses

Over the 5-year time period from April 1, 2012, to March 31, 2017, 101 PPP patients (incidence rate of 0.93 per million) and 5979 PV patients (incidence rate of 54.93 per million) had an MRD code defining the index event and no PPP/PV records in the 5 preceding years (April 1, 2007, to March 31, 2012). Compared to these national estimates, the incidence rates were higher in Alberta (PPP: 1.78 per million; PV: 158.84 per million) and Ontario (PPP: 1.19 per million; PV: 56.70 per million) due to mandatory reporting of visits to the ED (Ontario and Alberta) and outpatient clinics (Alberta).

Table III presents the baseline characteristics of our incident cohorts at index. There were no statistically significant differences between our cohorts in terms of the proportion of female patients (52.5% for PPP vs 45.3% for PV; *P* < .15) or age (mean [SD] of 49.8 [17.8] years for PPP vs 48.3 [17.3] years for PV; *P* = .38). There were no statistically significant differences in any of the comorbidities between PPP and PV.

**Table III:** Incidence analysis: baseline characteristics

	PPP (*N* = 101)	PV (*N* = 5979)	*P*-value
Female sex	53 (52.5%)	2711 (45.3%)	.15
Mean age (SD)	49.8 (17.8)	48.3 (17.3)	.38
Age categories, years			.16
18-25	14 (13.9%)	567 (9.5%)	
26-35	15 (14.9%)	984 (16.5%)	
36-45	9 (8.9%)	997 (16.7%)	
46-55	24 (23.8%)	1199 (20.1%)	
56-65	24 (23.8%)	1107 (18.5%)	
66-75	7 (6.9%)	704 (11.8%)	
≥75	8 (7.9%)	421 (7.0%)	
Province			.01
Alberta	28 (27.7%)	2505 (41.9%)	
Ontario	65 (64.4%)	3092 (51.7%)	
British Columbia, Saskatchewan, Manitoba, Nova Scotia, New Brunswick, Prince Edward Island, Newfoundland, and all 3 Territories	8 (7.9%)	382 (6.4%)	
Co-morbidities			
Hypertension	12 (11.9%)	756 (12.6%)	.82
Diabetes	10 (9.9%)	514 (8.8%)	.64
Cigarette smoking	5 (5.0%)	258 (4.3%)	.76
Hyperlipidemia	ND	147 (2.5%)	.76
Myocardial infarction	ND	94 (1.6%)	.74
Stroke	ND	45 (0.8%)	.38
Obesity	ND	145 (0.8%)	.35
Sleep apnea	ND	79 (1.3%)	.77
Depression	6 (5.9%)	307 (5.1%)	.72
Anxiety	ND	96 (1.67%)	.62
Thyroid disorders	ND	120 (2.0%)	.50
Atopic dermatitis	ND	68 (1.1%)	.09
Gout	ND	119 (2.0%)	.15
Psoriatic arthritis	ND	36 (0.6%)	.62

*ND*, Non disclosable due to cell count less than 5; *PPP*, palmoplantar pustulosis; *PV*, psoriasis vulgaris; *SD*, standard deviation.

The index event defining a PPP or PV incident case was mostly related to an ED visit (74% for PPP vs 77% for PV; *P* = .65). Due to the higher mean (SD) number of hospitalizations associated with PPP (0.16 [0.37] for PPP vs 0.02 [0.14] for PV; *P* < .01), the mean (SD) cost associated with the PPP index event was higher than the PV index event ($1070 [$2707] vs $275 [$1049]; *P* < .01). Table IV presents the details associated with the index visit, while Supplementary Table III, available via Mendeley at https://data.mendeley.com/datasets/9fnfyys96m/1 presents the results of the regression analyses. As shown in Table V, the cumulative 3-year mean (SD) MRD costs for PPP ($1078 [$2705]) were higher than PV ($503 [$2267]) (*P* < .01). However, the 3-year cumulative cost for incident cases was lower for PPP compared to PV when all healthcare resource use were included independently of the reasons leading to the admissions/visits ($6341 [$10,041] vs $10,109 [$30,212]; *P* < .01).

**Table IV:** Incidence analysis: mean (standard deviation) healthcare resource utilization and costs associated with index event

	PPP (*N* = 101)	PV (*N* = 5979)	*P*-value∗
Number of hospitalizations per case	0.16 (0.37)	0.02 (0.14)	.01†
Number of days in hospital for those with inpatient stay	8.0 (5.6)	6.2 (6.7)	.45
Cost of hospitalization for those with inpatient stay	$5802 ($4501)	$5217 ($5224)	.83
Number of ED visits per case	0.74 (0.44)	0.77 (0.42)	.65†
Cost of ED visit for those with ED visit	$157 ($62)	$160 ($70)	.88
Number of clinic and other NACRS visits per case	0.10 (0.30)	0.21 (0.41)	.01†
Cost of clinic and other NACRS records for those with a visit	$346 ($573)	**$**204 ($193)	.01
Total cost (1 + 2 + 3): Mean (SD)	$1070 ($2707)	$275 ($1049)	<.01

*ED*, Emergency department; *NACRS*, National Ambulatory Care Reporting System; *PPP*, palmoplantar pustulosis; *PV*, psoriasis vulgaris; *SD*, standard deviation.

∗ *P*-values based on multivariable generalized linear models adjusting for baseline characteristics (age, sex, and comorbidities).

† *P*-values based on multivariable logistic regressions adjusting for baseline characteristics as individuals had 0 or 1 event.

**Table V:** Incidence analysis: 3-year mean (standard deviation) healthcare resource utilization and costs associated post index event—most responsible diagnosis (MRD)∗ versus all-cause admissions

	Most responsible diagnosis (MRD) admissions			All-cause admissions		
	PPP (*N* = 101)	PV (*N* = 5979)	*P*-value∗	PPP (*N* = 101)	PV (*N* = 5979)	*P*-value†
Number of hospitalizations per case	0.16 (0.37)	0.03 (0.20)	<.01	0.72 (1.10)	0.62 (1.58)	.56
Number of hospitalizations for those with at least 1 hospitalization	1.00 (0.00)	1.12 (0.42)	.59	1.74 (1.06)	2.29 (2.33)	.06
Number of days in hospital for those with inpatient stay	8.00 (5.62)	6.62 (9.44)	.60	4.64 (3.39)	7.74 (12.74)	<.01
Cost of hospitalization for those with inpatient stay	$5802 ($4501)	$5530 ($6518)	.95	$5233 ($3688)	$9560 ($16,015)	<.01
Number of ED visits per case	0.76 (0.47)	0.88 (0.69)	.25	5.08(5.65)	5.89 (12.43)	.33
Number of ED visits amongst those with at least 1 ED visit	1.03 (0.16)	1.13 (0.56)	.39	5.31 (5.80)	6.53 (13.00)	.10
Cost of ED visit for those with ED visit	$160 ($59)	$163 ($66)	.64	$249 ($133)	$256 ($144)	.67
Number of clinic and other NACRS visits per case	0.13 (0.44)	1.0 (8.08)	<.01	3.14 (13.00)	6.34 (22.72)	.03
Number of clinic and other NACRS visits amongst those with at least 1 visit.	1.30 (0.67)	4.71 (16.99)	.01	7.37 (19.25)	12.21 (30.25)	.04
Cost of clinic and other NACRS visits for those with a visit	$346 ($573)	$204 ($191)	.01	$616 ($723)	$529 ($583)	.47
Total cost (1 + 2 + 3)	$1078 ($2705)	$503 ($2267)	<.01	$6,341 ($10,041)	$10,109 ($30,212)	<.01

*ED*, Emergency department; *NACRS*, National Ambulatory Care Reporting System; *PPP*, palmoplantar pustulosis; *PV*, psoriasis vulgaris; *SD*, standard deviation.

∗ The most responsible diagnosis (MRD) is the diagnosis (eg, PPP) determined to have been responsible for the greatest portion of the patient’s length of stay.

† *P*-values based on multivariable generalized linear models adjusting for baseline characteristics (age, sex, and comorbidities).

Similar to our prevalent mortality analysis, there were no statistically significant differences in the 3-year all-cause inpatient mortality following the index event. Due to small cells, the 3-year all-cause inpatient mortality for the PPP cohort could not be disclosed. Three-year all-cause inpatient mortality was 2.1% for PV incident cases. Supplementary Table IV (3-year costs) and Supplementary Table V (3-year all-cause inpatient mortality) present the details of multivariable regression models adjusting for baseline characteristics, which are available via Mendeley at https://data.mendeley.com/datasets/9fnfyys96m/1.

## Discussion

The results of these analyses using Canadian data on hospitalization and visits to the ED and outpatient clinics makes several contributions to the emerging literature on PPP. Due to a mandatory reporting of ED and hospital-/community-based clinics in Alberta compared to the other Canadian provinces/territories, the prevalence and incidence rates of PPP were the highest in Alberta at 3.45 per million individuals and 1.78 per million individuals, respectively. One of the key findings of this study is that the PPP MRD costs were higher than PV MRD costs by a factor ranging from 2.1 (incidence analysis) to 2.4 (prevalence analysis). However, the PPP cohort had lower costs than the PV cohort when all-cause admissions were considered in both the incident and prevalent approaches. These results highlight the need to document the economic burden directly attributed to the condition under study (eg, MRD cost) versus the burden associated with complications or comorbidities associated with PPP and PV. Our results showed that only a small part of the healthcare resource use and associated costs were attributable to an MRD of PPP or PV when compared to all-cause admissions. Another important finding of our study is that we did not find any difference in all-cause inpatient mortality between PPP and PV in our prevalent and incident analyses. Finally, in addition to our prevalent analysis, we undertook an incident analysis in which PPP patients were followed for 3 years after the index event defined by an MRD of PPP. To our knowledge, no other studies have taken an incidence approach when studying PPP.

Compared to an Ontario-specific study in Canada which identified 132 hospital and ED records with a PPP diagnostic code over a 10-year period,^6^ our results for Ontario (data not shown) are similar. However, we also included all other Canadian provinces/territories excluding Quebec, while providing additional insights on patient characteristics and the burden of PPP in Canada. It is difficult to compare our study findings with the limited international literature on PPP due to differences in study design, data availability, or health care settings. Since we only had access to hospitalizations and visits to the ED and outpatient clinics, our prevalence numbers are lower than those reported in other studies in which authors had also access to physician and pharmacy data.^2^ It is also difficult to compare our cost figures with the U.S. study which also included physician and pharmacy costs.^5^ Since the U.S. study did not provide the cost figures for the inpatient and ED use, we were not able to compare our hospitalization/ED cost results.

Several limitations associated with our study should be noted when interpreting the results. First, similar to other studies relying on administrative data, there is a possibility of miscoding or underreporting the conditions under study. For example, while it was assumed that healthcare resource use with a diagnostic code of PPP will be those from genuine PPP patients, some PPP patients may have not received a PPP diagnostic code. A second limitation is related to the data capture of the ED and hospital-/community-based outpatient clinic data. As opposed to hospitalizations, reporting of ED visits is only mandatory in a few Canadian provinces while reporting of hospital- and community-based outpatient clinic visits is only mandatory in Alberta. To address this issue, we reported prevalence and incidence rates for Ontario (mandatory reporting of ED visits) and Alberta (mandatory reporting of ED and outpatient clinic visits). Third, we did not have any information on physician and pharmacy data or mortality in the community. As a result, this study underestimates the true impact of PPP in Canada. Finally, since administrative data were used, we did not have access to quality-of-life data to document the burden of PPP on health-related quality of life. We also did not have any information on work/school productivity losses or out of pocket expenditures incurred by PPP patients and their caregivers. This is left for future research.

## Conclusions

Our study results indicate that PPP patients incurred significantly higher costs than PV patients when healthcare resource utilization was due to an MRD of PPP or PV but not when all cause health care resource use was considered. We also did not find any difference in all-cause inpatient mortality between PPP and PV.

## Conflicts of interest

McMaster University (Jean-Eric Tarride as Principal Investigator) has received a grant from Boehringer Ingelheim for this research. JET has received honoraria from AbbVie, Amgen, AstraZeneca, Bayer, Edwards Lifesciences, Janssen, Lilly, Leo Pharma, Merck, Novartis, Novo Nordisk, Pfizer, Roche, and Takeda outside of the scope of this paper. Vimal H. Prajapati has been an advisor, consultant, and/or speaker for AbbVie, Actelion, Amgen, Aralez, Arcutis, Arena, Aspen, Bausch Health, Boehringer Ingelheim, Bristol Myers Squibb, Celgene, Cipher, Concert, Eli Lilly, Galderma, GlaxoSmithKline, Homeocan, Incyte, Janssen, LEO Pharma, L’Oreal, Medexus, Novartis, Pediapharm, Pfizer, Sanofi Genzyme, Sun Pharma, Tribute, UCB, and Valeant; he has also served as an investigator for AbbVie, Amgen, Arcutis, Arena, Asana, Bausch Health, Boehringer Ingelheim, Bristol Myers Squibb, Celgene, Concert, Dermavant, Dermira, Eli Lilly, Galderma, Incyte, Janssen, LEO Pharma, Nimbus Lakshmi, Novartis, Pfizer, Regeneron, Reistone, Sanofi Genzyme, UCB, and Valeant. Charles Lynde has been a speaker and/or consultant to AbbVie, Altius, Amgen, Aralez, Arcutis, Bausch Health, Bayer, Boehringer Ingelheim, Bristol Myers Squibb, Celgene, Cipher, Dermavant, Devonian, Eli Lilly, Fresnius Kabi, Galderma, GSK, Innovaderm, Intega Skin, Janssen, Kyowa Kirin, La Roche Posay, LEO Pharma, L'Oreal, Medexus, MedX, Merck, Novartis, P&G, Pediapharm, Pfizer, Regeneron, Roche, Sanofi Genzyme, Sandoz, Sentrex, TEVA, Tribute, UCB, Valeant, Viatris, and Volo Health; he has also been a principal investigator for AbbVie, Acleryin, Amgen, Aralez, Arcutis, Bausch Health, Bayer, Boehringer Ingelheim, Bristol Myers Squibb, Celgene, Cipher, Concert, Dermavant, Devonian, Eli Lilly, Galderma, GSK, Innovaderm, Janssen, Kyowa Kirin, LEO Pharma, L'Oreal, Merck, MoonLake, Pediapharm, Pfizer, Regeneron, Roche, Sanofi Genzyme, Tribute, UCB, and Valeant. Gord Blackhouse has no conflicts of interest to disclose.
